# Ginkgo biloba extract protects human melanocytes from H_2_O_2_‐induced oxidative stress by activating Nrf2

**DOI:** 10.1111/jcmm.14393

**Published:** 2019-05-31

**Authors:** Shaolong Zhang, Xiuli Yi, Xin Su, Zhe Jian, Tingting Cui, Sen Guo, Tianwen Gao, Chunying Li, Shuli Li, Qian Xiao

**Affiliations:** ^1^ Department of Dermatology Xijing Hospital, Fourth Military Medical University Xi’an China

**Keywords:** EGb761, Melanocyte, Nrf2, Oxidative stress, Vitiligo

## Abstract

Vitiligo is a common skin depigmenting disorder characterized by the loss of functional melanocytes. Its pathogenesis is complicated and oxidative stress plays a critical role in the development of vitiligo. Thus, antioxidant therapy is a promising therapeutic strategy to prevent or even reverse the progression of depigmentation. Ginkgo biloba extract EGb761 has been confirmed to have protective effects on neurons against oxidative stress. Notably, several clinical trials have shown that patients with stable vitiligo achieved repigmentation after taking EGb761. However, the exact mechanism underlying the protective effects of EGb761 on melanocytes against oxidative stress has not been fully elucidated. In the present study, we found that EGb761 effectively protected melanocytes against oxidative stress‐induced apoptosis and alleviated the excessive accumulation of reactive oxygen species (ROS) and lipid peroxidation by enhancing the activity of antioxidative enzymes. Furthermore, the antioxidative effect of EGb761 was achieved by activating Nrf2 and its downstream antioxidative genes. In addition, interfering Nrf2 with siRNA abolished the protective effects of EGb761 on melanocytes against oxidative damage. In conclusion, our study proves that EGb761 could protect melanocytes from H_2_O_2_‐induced oxidative stress by activating Nrf2. Therefore, EGb761 is supposed to be a potential therapeutic agent for vitiligo.

## 1 INTRODUCTION

Vitiligo is a multifactorial disorder of skin characterized by the appearance of white patches, and its pathogenesis has not been fully elucidated. Previous studies have shown that both intrinsic and extrinsic factors contribute to the development of vitiligo.^1^ Among the etiological factors, oxidative stress can directly disturb melanin metabolism and undermine melanocyte survival through excessive accumulation of cytotoxic hydrogen peroxide.^2^, ^3^ Furthermore, oxidative stress could induce autoimmune T cell response that targets melanocytes in vitiligo pathogenesis.^3^ Therefore, antioxidants have been applied in the treatment of vitiligo.

Ginkgo biloba extract (EGb761) is a well‐known antioxidant and has shown remarkable therapeutic effects in a variety of oxidative stress‐related diseases, such as brain stroke, cardiovascular disease and Alzheimer's disease.^4^, ^5^, ^6^ Previous studies have demonstrated that EGb761 could prevent hippocampus neurons from oxidative damage by enhancing antioxidative ability of neurons, improving the microenvironment for nerve regeneration and promoting the proliferation of nerve cells.^4^, ^6^, ^7^ It is known that melanocytes originate from neural crest, so it has raised concerns about how EGb761 affects the growth of melanocyte cell lines in recent years. One study reported that EGb761 could protect melanocytes against oxidative stress by activating antioxidase and inhibiting endoplasmic reticulum stress.^8^ However, some other studies have controversial findings that Ginkgo biloba or its principal components could induce the apoptosis of melanoma cells.^9^, ^10^ Notably, several clinical trials have illustrated that the application of Ginkgo biloba extract can prevent the onset and the progression of vitiligo, indicating its therapeutic potential in treating vitiligo.^11^ However, the exact mechanism underlying the effect of EGb761 on vitiligo remains unclear.

The transcription factor nuclear erythroid 2‐related factor (Nrf2), which governs the natural function of phase II detoxification and the expression of antioxidant enzymes, is a master regulator of cellular defence against oxidative stress.^12^ Under oxidative stress, Nrf2 disassociates from kelch‐like ECH‐associated protein 1 (Keap1) and translocates into nucleus, thus promoting the transcription of its downstream antioxidative genes, such as *HO‐1*, *NQO‐1* and *SOD2*, through Nrf2‐ARE pathway.^13^ Our previous study has shown that vitiligo melanocytes have defects in activating Nrf2‐ARE pathway, which impaired the ability of melanocytes to resist oxidative damage.^14^ Recent studies have demonstrated that several drugs can protect melanocytes from oxidative stress by enhancing Nrf2‐ARE pathway.^15^, ^16^ Given that EGb761 prevents the death of melanocytes under oxidative stress, we suggested that EGb761 could activate Nrf2 and protect melanocytes from oxidative damage.

By transfecting the E6 and E7 genes of human papilloma virus type 16 (HPV16) into normal human epidermal melanocytes, PIG1 is produced as a cell line with unlimited growth potential and normal melanocytic properties.^17^ Moreover, our previous studies have demonstrated that PIG1 cells have similar biological characteristics as primary normal human melanocytes under oxidative stress.^18^, ^19^ In the present study, we made use of PIG1 cells to evaluate the protective effects of EGb761 on melanocytes under oxidative stress and testify our hypothesis.

## 2 MATERIALS AND METHODS

### 2.1 Experimental reagents and antibodies

The following reagents were obtained from the indicated commercial sources. EGb761, hydrogenperoxide (H_2_O_2_), dimethyl sulfoxide (DMSO) and 4,5‐dimethylthiazol‐2‐yl‐2,5‐diphenyltetrazolium bromide (MTT) were obtained from Sigma‐Aldrich (MO, USA). The kits for examining superoxide dismutase (SOD), malondialdehyde (MDA), reactive oxygen species (ROS) and glutathione peroxidase (GSH‐Px) were purchased from Nanjing Jiancheng Bioengineering Institute (Nanjing, China). The antibodies against Nrf2, HO‐1, NQO1 and SOD2 were purchased from Cell Signaling Technology (Boston, MA, US).

### 2.2 Cell culture and treatment

PIG1 cells were cultured in Medium 254 (Cascade Biolo‐gics/Invitrogen, Portland, OR USA) supplemented with human melanocyte growth supplement (Cascade Biologics/Invitrogen), 5% foetal bovine serum (Invitrogen, CA, USA) and penicillin‐streptomycin antibiotic mix (Invitrogen) at 37 ℃ in the presence of 5% CO_2_. EGb761 was freshly prepared as a stock solution in DMSO and diluted with Medium 254 supplements (0.1% (v/v) DMSO). Dimethyl sulfoxide was present at equal concentrations in all groups. H_2_O_2_ was freshly prepared from a 30% stock solution prior to each experiment. The treatment concentration of H_2_O_2_ was 1.0mM and the treatment concentration of EGb761 was 100 μg/ml.

### 2.3 MTT assay

PIG1 cells were cultured in 96‐well microtitre plates for 24 hours at 37℃ and 5% CO_2_. After different treatments, the supernatant was flicked off and washed once. Twenty μL of MTT (5 mg/mL) in PBS solution was then added to each well, and the plates were further incubated for 4 hours. After this, 150 μL of DMSO was added into the wells to solubilize the crystals. Finally, the optical density (OD) was measured at a wavelength of 490 nm. Cell survival rates were expressed as folds of that of untreated group (control).

### 2.4 Measurement of antioxidant enzymes

PIG1 cells were treated with 100 μg/ml of EGb761 or vehicle control (DMSO). After treatment with H_2_O_2_ for 24 hours, cells were collected and centrifuged at 10 000 g for 20 minutes. The SOD and GSH‐Px contents in the supernatant were assessed spectrophotometrically with the corresponding kits. The protein concentrations were determined by the Bradford method. The levels of SOD and GSH‐Px were expressed as units per milligram protein.

### 2.5 Measurement of MDA levels

Malondialdehyde in cells was measured using commercial kits according to the manufacturer's recommendations. The absorbance of the supernatant was determined at 532 and 600 nm. Malondialdehyde levels were expressed as nmol/mg protein.

### 2.6 Measurement of intracellular reactive oxygen species

Reactive oxygen species was monitored using dihydrodichlorofluorescein (H2DCF) cell‐permeant probe according to the manufacturer's recommendations. Briefly, PIG1 cells from different groups were collected and washed with PBS and then incubated for 20 minutes in the dark at 37℃ in PBS containing 10 μmol/L of H2DCF. The level of ROS generated was measured using flow cytometry based upon the fluorescence intensity of DCF at 525 nm after excitation at 485 nm. The levels of ROS were expressed as units of fluorescence compared with that of untreated group (control).

### 2.7 Protein extraction and Western blot analysis

After the completion of respective treatments, PIG1 cells were harvested and cellular protein lysate was prepared using lysis buffer [20 mmol/L Tris–HCl, 120 mmol/L NaCl, 1.0% Triton X 100, pH 7.5, 10% glycerol, 2 mmol/L EDTA and protease inhibitor cocktail (Roche GmbH, Germany)] at 4°C for 30 min. After centrifugation at 10 000 g for 20 minutes, the supernatant was separated and stored at −80°C until use. Protein concentrations were determined using 1× Bradford protein assay kit according to the manufacturer's instructions (Bio‐Rad, Hercules, CA) with bovine serum albumin (BSA) as a standard.

For Western blot, equal amounts of protein lysates were separated in 10% sodium dodecyl sulphate‐polyacrylamide (SDS‐PAGE) gel electrophoresis. The gels were blotted onto a nitrocellulose membrane and incubated with the indicated antibodies. Blots were developed by enhanced chemiluminescence (ECL) according to the manufacturer's instructions. Actin was used as a loading control.

### 2.8 Quantitative polymerase chain reaction

Total RNA was extracted using the TRIzol Reagent (Invitrogen, Carlsbad, CA). RNA concentrations and purities were determined by measuring the absorbance at 260 nm. First‐strand cDNA was generated using SuperScript III first‐strand synthesis system as recommended by the manufacturer (Invitrogen). Reverse transcription was performed using 1 μg of total RNA in 12.5 μL of the solution containing 4 μL of 25 mM MgCl_2_, 4 μL of AMV reverse transcriptase 5 × buffer, 2 μL dNTP, 0.5 μL of RNase inhibitor, 1 μL of AMV reverse transcriptase and 1 μL of oligo dT primer, all of which were added with nuclease‐free water to make a final volume of 20 μL. Reaction system was run at 42°C for 50 minutes and 95°C for 5 minutes. Primers used for the reactions were purchased from Invitrogen and the sequences were as follows: Nrf2: forward, 5′‐CTTGGCCTCAGTGATTCTGAAGTG‐3′, reverse, 5′‐CCTgAGATGGTGACAAGGGTTGTA‐3′; HO‐1: forward, 5′‐CAGGAGCTGCTGACCCATGA‐3′; reverse, 5′‐AGCAACTGTCGCCACCAGAA‐3′. NQO1: forward, 5′‐GGTAGCGGCTCCATGTACTC‐3′, reverse, 5′‐CATCCTTCCAGGATCTGCAT‐3′; SOD2: forward, 5′‐ AACCTCACATCAACGCGCAGATC‐3′, reverse, 5′‐ CTCCTGGTACTTCTCCTCGGTGAC‐3′. Real‐time quantitative PCR (RT‐qPCR) was carried out in 20 μL of reaction buffer that included 10 μL of TaqMan Universal PCR Master Mix, 1 μL of primer and 9 μL of cDNA with the BIO‐BAD CFX96 Touch^TM^ Real‐Time qPCR system. The GAPDH normalized data were presented as the fold change in gene expressions in the treatment groups compared with control groups.

### 2.9 Statistical analysis

The statistical analyses were performed using spss 16.0 (SPSS Inc, Chicago, IL). All the values were presented as mean ± standard deviation (SD). Statistical analysis was performed by two‐tailed Student's *t* test or ANOVAs, followed by Bonferroni/Dunn post hoc comparisons. *P* < 0.05 was regarded statistically significant.

## 3 RESULTS

### 3.1 EGb761 improves cell viability and inhibits the production of ROS and lipid peroxidation in melanocytes under oxidative stress

**Figure 1:**
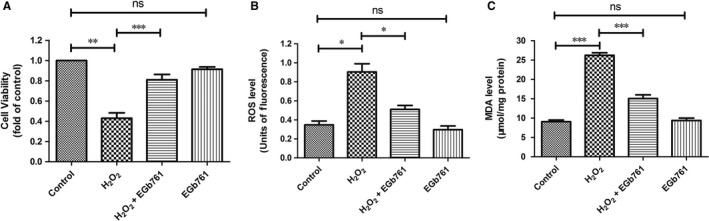
EGb761 protects PIG1 cells from H_2_O_2_‐induced apoptosis, intracellular ROS generation and lipid peroxidation. Cells were pretreated with EGb761 for 48 h, and then incubated with 1.0 mmol/L H_2_O_2_ for 24 h. Then, cell viability, cellular ROS activity and MDA levels were measured. A, cell viability measured by MTT assay. H_2_O_2_ reduced the cell viability and the pretreatment with EGb761 alleviated H_2_O_2_‐induced cell apoptosis. B, DCFH‐DA fluorescence of cells was measured by flow cytometry. The intracellular ROS level of PIG1 cells was increased under the treatment of H_2_O_2_, and the pretreatment with EGb761 reduced H_2_O_2_‐induced ROS production. C, Cellular MDA level was measured by TBA assay. The MDA level of melanocytes was increased under the treatment of H_2_O_2_, and the pretreatment with EGb761 reduced H_2_O_2_‐induced MDA generation. Three repeated experiments were made, and paired *t* test was used to make statistics. Data are presented as the mean ± SD, *P < 0.05, **P < 0.01

To examine whether EGb761 could protect melanocytes under oxidative stress, we built the oxidative stress model of melanocytes based on our previous studies, which was made by treating PIG1 cells with 1.0 mmol/L H_2_O_2_. Obviously, H_2_O_2_ reduced the cell viability by 0.57 folds (*P* = 0.012) and the pretreatment with EGb761 significantly alleviated H_2_O_2_‐induced cell apoptosis by 0.38 folds (*P* = 0.003) compared with control (Figure 1A). Then, we detected the intracellular ROS level by using ROS probes using flow cytometry and the activity of lipid peroxidation by measuring MDA level in H_2_O_2_‐treated PIG1 cells. Compared with the control group, the intracellular ROS level of PIG1 cells was significantly increased by 1.57 folds under the treatment of H_2_O_2_. Notably, the pretreatment with EGb761 effectively reduced H_2_O_2_‐induced ROS burden by nearly 30%, although a single treatment with EGb761 did not change intracellular ROS level in PIG1 cells (Figure 1B). Moreover, EGb761 slightly reduced the level of MDA in PIG1 cells and further ameliorated about 42% of the increased level of MDA induced by H_2_O_2 _(Figure 1C).

### 3.2 EGb761 restores the activity of SOD and GSH‐Px in melanocytes under oxidative stress

**Figure 2:**
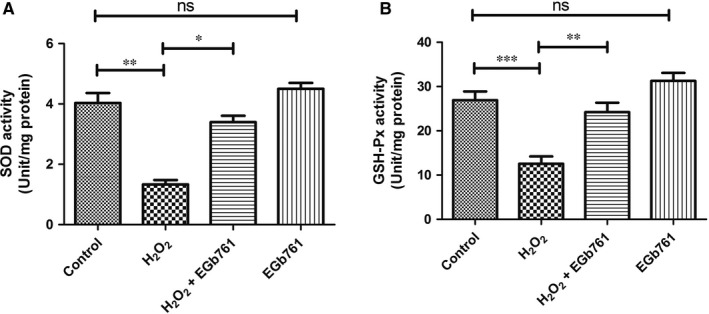
Effect of EGb761 on the activity of antioxidant enzymes. Cells were pretreated with EGb761 for 48 h, and then incubated with 1.0 mmol/L H_2_O_2_ for 24 h. The activity of cellular SOD (A) and GSH‐Px (B) were measured. H_2_O_2_‐treated melanocytes displayed impaired activity of SOD and GSH‐Px, and the pretreatment with EGb761 restored the inhibited enzyme activity caused by H_2_O_2_. Three repeated experiments were made, and paired *t* test was used to make statistics. Data are presented as the mean ± SD, *P < 0.05, **P < 0.01, ***P < 0.001

Previous studies have indicated that the imbalance in redox state is involved in the pathogenesis of vitiligo.^14^, ^20^, ^21^ As EGb761 effectively reduced ROS level in H_2_O_2_‐treated PIG1 cells, we speculated that the effect of EGb761 could enhance the activity of antioxidative enzymes in melanocytes. To testify this, the activity of two main antioxidative enzymes including SOD and GSH‐Px were measured in H_2_O_2_‐treated melanocytes. As expected, H_2_O_2_‐treated melanocytes displayed impaired activity of SOD and GSH‐Px by 59% and 57%, respectively, while the pretreatment with EGb761 restored almost half of the inhibited activity of the two enzymes caused by H_2_O_2_ (Figure 2A,B).

### 3.3 EGb761 activates Nrf2‐ARE signaling pathway in melanocytes under oxidative stress

**Figure 3:**
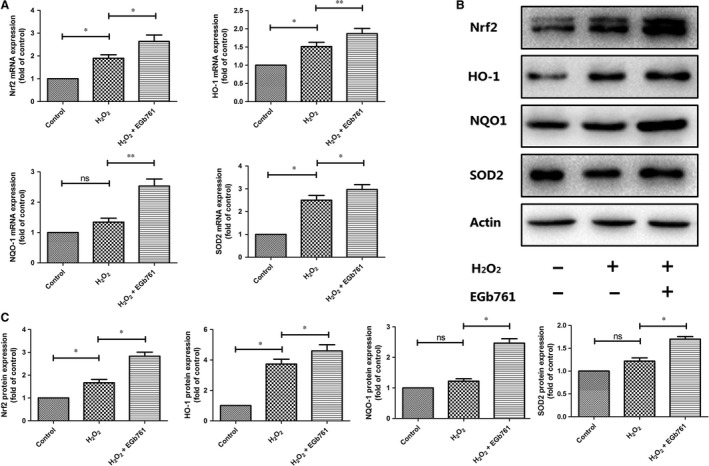
Effect of EGb761 on Nrf2 and HO‐1 protein and gene expression. Cells were pretreated with EGb761 for 48 h, and then incubated with 1.0 mmol/L H_2_O_2_ for 24 h. The mRNA levels of NQO1, SOD2, Nrf2 and HO‐1 were semi‐quantified by RT‐PCR analyses (A). Actin was used as a loading control. The mRNA expressions of Nrf‐2, NQO1, SOD2 and HO‐1 in EGb761 pretreatment group were significantly increased compared with single H_2_O_2_ treatment group (the increased folds as 0.7, 1.2, 0.4 and 0.4, respectively; *P* = 0.037, 0.008, 0.033 and 0.007 respectively). Total cell lysates were prepared and subjected to Western blot analysis to monitor the expression levels of antioxidant proteins including NQO1, SOD2, Nrf2 and HO‐1 (B and C). Actin served as an internal control. The protein expression levels of Nrf2 and its downstream antioxidative genes including *NQO1*, *SOD2* and *HO‐1* increased slightly in H_2_O_2_‐treated group, and they were further increased significantly in EGb761‐pretreated group (the increased folds compared with single H_2_O_2_‐treated group as 1.2‐fold, 1.3‐fold, 0.5‐fold and 0.3‐fold, respectively; *P* = 0.029, 0.03, 0.04 and 0.027 respectively). Three repeated experiments were made, and paired *t* test was used to make statistics. Data are presented as the mean ± SD, *P < 0.05, **P < 0.01

To further identify whether the protective effect of EGb761 on melanocytes under oxidative stress is dependent on Nrf2‐ARE pathway, the mRNA and protein levels of Nrf2 and its downstream genes were detected in PIG1 cells treated with H_2_O_2_ by qPCR and Western blot respectively. As is illustrated in Figure 3B,C, the protein levels of Nrf2 and its downstream antioxidative genes including *NQO1, SOD2* and *HO-1* were slightly increased in H_2_O_2_‐treated PIG1 cells, while they were further significantly increased in EGb761‐pretreated PIG1 cells. We further performed qPCR assays to confirm our results of Western blot analysis. (Figure 3A). Consistent with the changes of protein levels, the mRNA expressions of Nrf‐2, NQO1, SOD2 and HO‐1 in EGb761‐pretreated group were significantly elevated compared with the group under single treatment with H_2_O_2_ (with the increased folds ranging from 0.4 folds to 1.2 folds, *P* < 0.05).

### 3.4 Nrf2 knockdown diminishes the protective effect of EGb761 on melanocytes under oxidative stress

**Figure 4:**
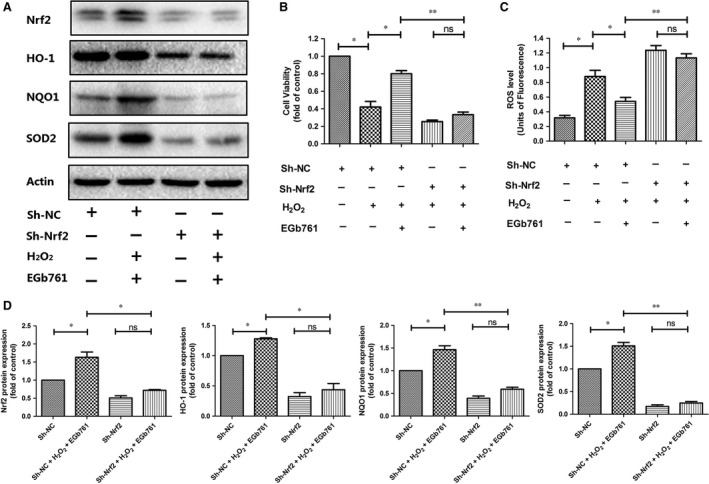
Silencing Nrf2 gene abolished the prevention made by EGb761. PIG1 cells were transfected with siRNA or empty vector for 36 h, followed by 48 h pretreatment with EGb761 at 100 μg/ml, and then subjected to H_2_O_2_ (1.0 mmol/L). The expressions of Nrf2, SOD2, HO‐1 and NQO1 were measured by Western blotting (A and D). The pretreatment with EGb761 increased the levels of Nrf2, SOD2, HO‐1 and NQO1 in si‐NC group, which was not observed in si‐Nrf2 group. The cell viability (B) and ROS level (C) of cells were determined respectively. Compared with single H_2_O_2_‐treated cells, the pretreatment with EGb761 significantly increased the number of viable cells in the si‐NC group, whereas the percentage of viable cells was further decreased and it failed to reverse in si‐Nrf2 group. The ability of EGb761 to inhibit ROS levels was also diminished in si‐Nrf2 group. Three repeated experiments were made, and paired *t* test was used to make statistics. Data are presented as the mean ± SD, *P < 0.05, **P < 0.01, ***P < 0.001

To further demonstrate the involvement of Nrf2 on the protective effect of EGb761 on melanocytes under oxidative stress, we established PIG1 cell lines with Nrf2 knockdown by using siRNA transfection. After the transfection with si‐Nrf2, the protein levels of Nrf2 and its downstream genes including *NQO‐1*, *HO‐1* and *SOD2* were reduced by 89%, 53%, 67% and 82% respectively (Figure S1). We further found that the pretreatment with EGb761 effectively increased the protein levels of Nrf2, SOD2, HO‐1 and NQO1 in PIG1 cells transfected with scrambled siRNA (si‐NC), which, however, could not be observed in the cells with Nrf2 knockdown (Figure 4A,D). Subsequently, the viability of PIG1 cells was measured by MTT assay after the transfection with si‐Nrf2 or si‐NC. Compared with the group under single treatment with H_2_O_2_, the pretreatment with EGb761 significantly increased the number of viable cells in si‐NC group (from 0.41‐fold change to 0.82‐fold change, *P* = 0.014), whereas the percentage of viable cells was further decreased (from 0.41‐fold to 0.24‐fold, *P* = 0.144) and it failed to rescue the decreased viable cells (from 0.24‐fold to 0.32‐fold, *P* = 0.184) in Nrf2 knockdown group (Figure 4B).The inhibitory effect of EGb761 on intracellular ROS level in PIG1 cells treated with H_2_O_2_ was also diminished after the transfection with si‐Nrf2 (Figure 4C). Collectively, these data obviously indicate that the cytoprotective effect of EGb761 on melanocytes under oxidative stress is achieved by activating Nrf2.

## 4 DISCUSSION

In the present study, we primarily found that EGb761 could prevent melanocyte death under oxidative stress, inhibit the accumulation of ROS and enhance the activity of antioxidants. Moreover, the protective effect of EGb761 on melanocytes under oxidative stress was dependent on the activation of Nrf2 and its downstream antioxidative genes including *NQO‐1*, *SOD2* and *HO‐1*. Therefore, EGb761 could be an effective agent in protecting melanocytes against oxidative damage.

Ginkgo biloba extract has been applicated as an antioxidant to treat several oxidative stress‐related diseases like Alzheimer's disease or acute ischaemic stroke.^4^, ^6^, ^22^ Oxidative stress plays a pivotal role in the pathogenesis of vitiligo,^23^ thus it has gained concerns about whether Ginkgo biloba extract has therapeutic effect on vitiligo. A previous study has suggested that EGb761 could protect melanocytes from H_2_O_2_‐induced oxidative damage via inhibiting endoplasmic reticulum stress.^8^ Other studies on melanoma have reported that EGb761 and its active component flavonoids could induce the apoptosis of several melanoma cell lines,^9^, ^10^ whereas EGb761 has no lethal effect on normal melanocytes^10^ and flavonoids could protect melanoma cell line B16F1 from oxidative stress‐induced DNA damage.^9^ Nevertheless, some clinical trials have achieved encouraging results. A double‐blind placebo‐controlled clinical trial that recruited 47 vitiligo patients showed that the administration of 40 mg of Ginkgo biloba TID could arrest the disease progression and induce repigmentation of most acrofacial vitiligo lesion.^24^ Another study found that 60 mg of Ginkgo biloba BID could decrease the disease severity in vitiligo patients.^25^

In the present study, we initially found that EGb761 showed no toxic effect on PIG1 cells (data not shown), which was consistent with previous studies.^10^ The patients with vitiligo have inferior capacity against oxidative stress due to the lower activity of SOD and GSH‐Px.^20^, ^21^ Our study showed that EGb761 could significantly restore the activity of SOD and GSH‐Px in PIG1 cells under H_2_O_2_ treatment. Moreover, EGb761 could alleviate the accumulation of intracellular ROS and MDA, the two oxidative stress indicators, in H_2_O_2_‐treated PIG1 cells, highlighting the therapeutic potential of EGb761 in vitiligo.

Our previous studies have confirmed that defective Nrf2‐ARE activation leads to impaired ability of melanocytes to resist oxidative stress damage, and enhancing Nrf2 activity is a potential therapeutic strategy for the treatment of vitiligo.^14^ In the present study, EGb761 effectively up‐regulated Nrf2 and its downstream antioxidative genes at both transcriptional and translational level. Besides, the knockdown of Nrf2 diminished the protective effect of EGb761 on melanocytes against oxidative stress. Collectively, these data strongly prove that the antioxidant effect of EGb761 on melanocytes is achieved by activating Nrf2.

EGb761 is widely used in treating cardiovascular, neurological, endocrinological and immunological disorders.^4^, ^5^, ^6^, ^7^ Furthermore, its safety and effectiveness in clinical use have been well recognized and its adverse effects are rare.^26^ A clinical study reported that vitiligo patients achieved repigmentation by using 40 mg of Ginkgo biloba extract TID with slight adverse effects like mild nausea.^24^ In the present study, 100 μg/ml of EGb761, which was lower than the concentration of EGb761 for treating other diseases, could effectively protect melanocytes against H_2_O_2_‐induced oxidative damage. Therefore, the application of this concentration would be safe to vitiligo patients.

In conclusion, EGb761 can protect melanocytes against oxidative stress damage through the activation of Nrf2‐ARE signaling pathway. Our study provides a new strategy in treating vitiligo with traditional Chinese medicine. As EGb761 is widely used in the treatment of oxidative stress‐related diseases with remarkable safety and tolerance, its application in the treatment of vitiligo is prospective.

## CONFLICT OF INTEREST

The authors declare they have no conflict of interest and are responsible for the contents of this report.
