# Supplementary figures and images for: Ginkgo biloba extract protects human melanocytes from H2O2‐induced oxidative stress by activating Nrf2
Source: J Cell Mol Med. 2019 May 31;23(8):5193–9. doi: 10.1111/jcmm.14393 (PMC6653340; doi:10.1111/jcmm.14393)

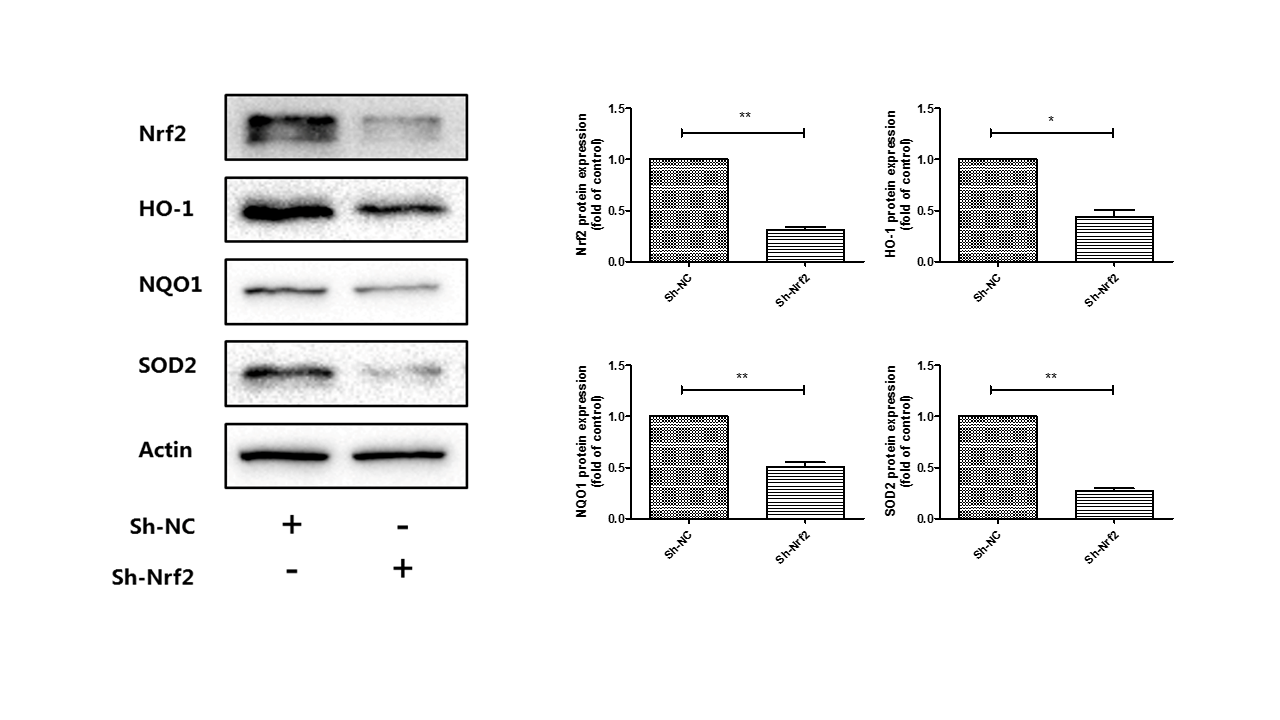

Supplement: Supplementary file 1 [file JCMM-23-5193-s001.tif]
