# Supplementary material for: Ginkgo biloba extract protects human melanocytes from H2O2‐induced oxidative stress by activating Nrf2
Source: J Cell Mol Med. 2019 May 31;23(8):5193–9. doi: 10.1111/jcmm.14393 (PMC6653340; doi:10.1111/jcmm.14393)
Supplement: Supplementary file 2 [file JCMM-23-5193-s002.docx]

Supplementary Figure 1. The interference efﬁciency of si-Nrf2 at protein levels. PIG1 cells were transfected with speciﬁc Nrf2 siRNA or null vector for 36 h. Subsequent western blotting assay showed that Nrf2-speciﬁc siRNA could efficiently knock down Nrf2 expression at protein level. Three repeated experiments were made, and paired t-test was used to make statistics. Data are presented as the mean ± SD, **P* < .05, ***P* < .01, ****P* < .001.
